# Prospective Cohort Study Identifies Medical Predictors of Treatment-Related Oral Toxicities in Oral and Oropharyngeal Cancer Patients

**DOI:** 10.3390/dj12040089

**Published:** 2024-04-01

**Authors:** Leticia Rodrigues-Oliveira, César Rivera, Xaviera A. López-Cortés, Milena Perez Mak, Ana Leticia Mores, Cesar Augusto Migliorati, Maria Cecília Querido de Oliveira, Natalia Rangel Palmier, Luiz Alcino Gueiros, Pablo Agustin Vargas, Thaís Bianca Brandão, Alan Roger Santos-Silva, Ana Carolina Prado-Ribeiro

**Affiliations:** 1 Oral Diagnosis Department, Piracicaba Dental School, University of Campinas (UNICAMP), Piracicaba 13414-903, Brazil; leticia.lro@gmail.com (L.R.-O.); analemores@gmail.com (A.L.M.); pavargas@unicamp.br (P.A.V.); carol_pr@yahoo.com.br (A.C.P.-R.); 2 Stomatology and Basic Biomedical Sciences Departments, Faculty of Health Sciences, Universidad de Talca (UTALCA), Talca 3460000, Chile; cesar.rivera.martinez@gmail.com; 3 Department of Computer Sciences and Industries, Catholic University of the Maule, Talca 3460000, Chile; xaviera.lopez.c@gmail.com; 4 Medical Oncology, Instituto do Câncer do Estado de São Paulo, Faculdade de Medicina da Universidade de São Paulo, São Paulo 01246-000, Brazil; milena.mak@hc.fm.usp.br; 5 Department of Oral and Maxillofacial Diagnostic Sciences, University of Florida College of Dentistry, Gainesville, FL 32603, USA; c.migliorati@dental.ufl.edu; 6 Dental Oncology Service, Instituto do Câncer do Estado de São Paulo, Faculdade de Medicina da Universidade de São Paulo, São Paulo 01246-000, Brazil; mcqdeoliveira@gmail.com (M.C.Q.d.O.); natpalmier@gmail.com (N.R.P.); thais.brandao@uol.com.br (T.B.B.); 7 Departamento de Clínica e Odontologia Preventiva, Universidade Federal de Pernambuco, Recife 50670-901, Brazil; lagueiros@gmail.com

**Keywords:** medical history, medical examination, dental care, comorbidity, oral cancer, oropharyngeal cancer

## Abstract

The dental treatment of patients with oral cavity and oropharyngeal squamous cell carcinoma (OOPSCC) may be challenging for dentists. This study aimed to characterize systemic changes in patients with OOPSCC undergoing dental treatment prior to cancer therapy, with a specific focus on laboratory assessments. The primary objectives included identifying potential adverse events, such as infections or bleeding, resulting from dental procedures. Additionally, the study aimed to correlate baseline patient characteristics with treatment-related toxicities. This was a prospective cohort study that included 110 OOPSCC patients referred to the Dental Oncology Service at São Paulo State Cancer Institute, Brazil, between November/2019 and December/2020. Comorbidities, sociodemographic data, medication in use, cancer treatment-related toxicities, and altered laboratory tests results were correlated. The most common comorbidities and altered laboratory results were hypertension, dyslipidemia, diabetes, as well as elevated levels of C-reactive protein, hemoglobin, and hematocrit. Toxicities exhibited a progressive pattern over time, encompassing oral mucositis (OM), xerostomia, dysphagia, dysgeusia, trismus, and radiodermatitis. No correlation between comorbidities and cancer treatment-related toxicities, a positive correlation between medications in use and OM, and a negative correlation between medications and dysgeusia were found. OM was associated with altered thyroxine (T4) and free thyroxine (FT4), calcium, urea, creatinine, alkaline phosphatase, and syphilis. Family income and housing were OM predictors. Altered T4/FT4/urea/calcium/alkaline phosphatase/creatinine/syphilis may be useful clinical predictors of OM. Despite the elevated prevalence of comorbidities and abnormal laboratory findings, dental treatment prior to cancer treatment yielded no adverse events.

## 1. Introduction

The dental treatment of patients diagnosed with oral cavity and oropharyngeal squamous cell carcinoma (OOPSCC) poses a challenge for dentists. It requires an individualized treatment plan that is based on the patient’s dental and medical history, cancer stage, treatment modalities, prognosis, and hematological, physical, and nutritional status [1,2,3].

It is recommended that the dental care for OOPSCC cancer patients be performed and completed before the onset of the oncologic treatment, especially for patients undergoing chemotherapy (CT), radiotherapy (RT), or chemoradiotherapy (CRT). Dental treatment must prioritize the removal of oral foci of infection that can interrupt the cancer treatment and impair prognostic outcomes. In this sense, periodontal, restorative, endodontic, and surgical procedures should be performed based on clinical and radiographic assessments, considering the oncologic treatment plan and schedule [1,2].

Current evidence shows that adequate oral care and proper dental treatment before the oncologic treatment is associated with fewer oral and systemic infections by minimizing the incidence, severity, and duration of oral toxicities, such as oral mucositis (OM), hyposalivation, dysgeusia, dysphagia, radiation caries, soft tissue necrosis, trismus, and osteoradionecrosis, among others. It also contributes significantly to the success of the cancer treatment, avoiding interruptions, reducing overall costs, and improving the patients’ quality of life and prognosis (QoL) [3,4].

Despite the undeniable benefits of initiating dental care promptly upon cancer diagnosis for OOPSCC patients, concerns among healthcare professionals persist, hindering the delivery of comprehensive oral care. Previous studies, such as those by Epstein et al. (2014) [1] and McGuire (2003) [5], underscore the clinical challenges associated with dental care in medically complex populations undergoing cancer treatment. Collaboration between physicians and dental professionals is imperative, as highlighted by the works of Lawrence et al. (2013) [6], emphasizing the need for knowledge, experience, and integration within the oncology team to ensure the best appropriate oral and dental care. Recognizing the unique complexities of oral care in oncology, including understanding cancer diagnosis, treatment plans, and post-therapy complications, adds another layer of difficulty. The identification of experienced and knowledgeable dental providers in the community further compounds the challenge, as noted by Epstein et al. (2014) [1]. This multidimensional perspective reinforces the critical nature of timely and collaborative oral care in optimizing outcomes for OOPSCC patients.

Although there are several dental care protocols for patients diagnosed with OOPSCC before cancer treatment [1,2,3], none of them considers systemic changes and the underlying medical conditions of the patient. Hence, this prospective cohort study aims to delineate, via laboratory assessments, systemic alterations in patients with OOPSCC who undergo dental treatment before starting cancer therapy. This investigation also explores potential adverse events arising from dental procedures, such as infection and bleeding. Furthermore, this study seeks to establish correlations between comorbidities, sociodemographic information, medication use, and laboratory changes and the occurrence and severity of toxicities in the head and neck (H and N) region.

## 2. Materials and Methods

This prospective cohort study recruited patients diagnosed with OOPSCC who were set to undergo oncologic treatment (i.e., surgery, CT, RT, or CRT) and were referred to the Dental Oncology Service at the São Paulo State Cancer Institute (ICESP), São Paulo, Brazil, for dental treatment. Patients were recruited between November/2019 and December/2020. Ethical approval was obtained from the National Human Research Ethics Committee (CAAE: 23671019.1.1001.5418). All participants provided written informed consent. The study was conducted following the Declaration of Helsinki and the strengthening the reporting of observational studies in epidemiology (STROBE) statement [7].

Fully or partially dentate OOPSCC patients over 18 years of age who were able to provide written informed consent were included in the study, regardless the cancer treatment modality.

Patients with recurrent OOPSCC that underwent a previous treatment, those who did not perform the blood tests prescribed, and cases in which a patient’s data were not fully available from the electronic medical record system were excluded from the study.

### 2.1. Sociodemographic and Clinicopathological Characteristics

Patients’ characteristics, including gender, ethnicity, age, habits (smoking, drinking and use of illicit drugs), years of education, current marital status, housing status, average monthly income, medical history, and medications in use were collected during a standardized in-person interview. Tumor location, cancer staging (TNM, 8th edition) [8,9], p16 status, proposed cancer treatment protocol, the Eastern Cooperative Oncology Group (ECOG) [10] and the Karnofsky performance status (KPS) [11] scores were extracted from the institutional electronic medical record system.

When indicated, all patients underwent dental procedures, including oral surgery, periodontal, endodontic, and restorative treatments. During dental treatment conditioning protocols, patients were followed up to evaluate possible treatment complications, such as infection and persistent bleeding, among others.

### 2.2. Anthropometric, Pulse Oximetry, Blood Pressure and Body Temperature Measurements

Height, weight, oxygen saturation levels, heart rate (HR), blood pressure (BP), and temperature were measured at the screening appointment. Height (cm) and weight (kg) were measured using a standardized scale with a stadiometer. Body mass index (BMI) was calculated as body weight divided by height squared (kg/m^2^) [12].

A pulse oximeter was used to measure oxygen saturation levels and HR [13]. BP was measured using an electronic BP monitor on the right upper arm, and the participants were asked to rest in a sitting position for 5 min before the measurement [14]. Body temperature was measured with a digital thermometer in the axillary region.

### 2.3. Laboratory Tests

Venous blood was collected by a trained nurse using standard methods and sent to the hospital’s laboratory for a complete blood count (CBC) and a standard blood chemistry panel (Supplementary Table S1). The following exams were performed:

– CBC with differential (erythrocytes, hemoglobin, hematocrit, mean corpuscular volume, mean corpuscular hemoglobin, mean corpuscular hemoglobin concentration, erythrocyte distribution width, erythrocyte distribution width (standard deviation, SD), erythroblasts, platelets, mean platelet volume, leukocytes, neutrophils, eosinophil, basophil, lymphocytes, and monocytes);
– Basic electrolyte panel (sodium, potassium, chloride, iron, creatinine, urea, glucose, magnesium);
– Metabolic panel ((calcium, bilirubin (total, direct, and indirect)), alkaline phosphatase, AST (aspartate aminotransferase), ALT (alanine aminotransferase, gamma-glutamyl);
– Lipid panel ((total cholesterol, high-density lipoprotein (HDL), triglycerides, low-density lipoprotein (LDL), non-high-density lipoprotein (non-HDL), VLDL (very-low-density lipoprotein));
– Thyroid function (TSH, T3, T4, FT4);
– Glycated hemoglobin (hemoglobin A1c);
– Coagulation assay [prothrombin time (PT), international normalized ratio (INR), activated partial thromboplastin time (aPTT)];
– Human immunodeficiency virus (HIV);
– Hepatitis B and C;
– 25-Hydroxy vitamin D;
– C-reactive protein (CRP);
– Syphilis.

### 2.4. Treatment-Related Head and Neck Toxicities

Systemic changes and abnormal laboratory results undertaken before the initiation of oncologic treatment were assessed and correlated with the treatment-related toxicities for patients submitted to RT or CRT. Included patients were clinically evaluated by a trained dentist for OM, radiodermatitis, dysgeusia, and dysphagia outcomes following the common terminology criteria for adverse events (NCI, version 4.0, 2010) [15], graded 0–4, at days 5/10/15/20/25/30/33–35 of radiation therapy. Additionally, oral candidiasis (OC), xerostomia, and trismus were evaluated qualitatively [16].

### 2.5. OHIP-14

The validated Brazilian Portuguese version of the Oral Health Impact Profile (OHIP-14) questionnaire was applied at the first appointment. It comprises 14 items, including functional limitation, physical pain and disability, psychological discomfort and disability, social disability, and handicap. The responses were classified using a Likert scale with five options, ranging from “never” (0) to “very often” (4) [17,18].

### 2.6. Statistical Analysis

Continuous variables such as clinicopathological, sociodemographic, anthropometric, pulse oximetry, BP, temperature measurements, and OHIP-14 data, were summarized using mean values and standard deviations; categorical variables analysis evaluated frequencies and percentages. For the laboratory results, the percentage of exams altered, mean values, and standard deviations were evaluated.

The chi-square test and Fisher’s exact test were applied to evaluate whether curative or palliative treatment modalities were influenced by tumor location, cancer staging, p16 status, and smoking and drinking habits. Pearson’s correlation test was used to evaluate the association between medication in use and treatment-related toxicities (OM, dysgeusia, dysphagia, xerostomia, radiodermatitis, trismus, and OC). All treatment toxicities on treatment days (D) 5/10/15/20/25/30/33–35 were associated with the laboratory findings. Additionally, using the two-tailed Spearman correlation test for a non-normal distribution, each toxicity outcome was evaluated when an altered laboratory exam was observed more than four different times on evaluation days, therefore representing a greater chance of a real association and a possible correlation. 

In all analyses, a *p*-value < 0.05 was considered statistically significant.

The worst OM score (group 1: grades 1/2 and group 2: grades 3/4) was correlated with the full data set. A continuous dataset (variables in their numerical forms), a discrete dataset (coded data), and a full dataset including all data were used. A biomarker analysis was performed to assess accuracy, sensitivity, specificity, positive predictive value, negative predict value, and area under the ROC curve. A heat map was presented to evaluate the clinical predictors of OM.

## 3. Results

### 3.1. Sociodemographic and Clinicopathological Characteristics

A total of 484 H and N cancer patients were referred to the Dental Oncology Service for dental treatment and evaluation within the study period. Among these, 110 (22.7%) met the eligibility criteria and 368 (76%) were excluded due to edentulism, an H and N cancer diagnosis other than OOPSCC, a second OOPSC cancer diagnosis, or for declining participation in the study. Six (1.3%) patients were further excluded for not undergoing the bloodwork prescribed. 

Most patients were male (n = 85; 77.3%), identified their ethnicity as white (n = 48; 43.6%) or brown (n = 48; 43.6%), and reported a history of tobacco (n = 94; 85.4%) and alcohol consumption (n = 94; 85.4%). Ages ranged from 23 to 83 (mean 57.32) years. Participants had an education level of 4 to 7 years (n = 42; 38.2%), were married/living with a partner (n = 46; 41.8%), owned a house (n = 72; 65.4%), and the most prevailing monthly income was 1045 Brazilian reals (BRL) (approximately $235.00 US dollars (USD)—2022 values) (n = 38; 34.5%). Fifty-four percent of the patients (n = 60) were diagnosed with oropharyngeal squamous cell carcinoma, while 45.5% (n = 50) had an oral tumor. Advanced disease was frequently observed (stage III/IV n = 85; 77.3%). The summaries of the sociodemographic and clinicopathological characteristics are shown in Table 1.

### 3.2. ECOG and KPS Performances

Most patients (n = 72; 65.5%) scored a 1 on the ECOG performance status, and 56 patients (50.9%) scored 90% on the KPS scale.

### 3.3. Referral Patterns and Oncologic Treatment Plans

Eighty patients (72.8%) were referred for dental treatment before the start of RT, 24.5% (n = 27) before surgery, and 2.7% (n = 3) during CT. Most of them (n = 82; 75.5%) were treated with curative intent, and the most performed treatment modality was CRT (n = 25; 30.5%) (Table 1). Early-stage tumors (I/II) were more frequently treated with a curative intent (*p* = 0.0).

### 3.4. Self-Reported Medical Conditions and Medication in Use

Forty patients (36.4%) did not report having any medical conditions other than cancer, 31 (44.3%) reported having one, and 39 (55.7%) patients had two or more underlying medical conditions.

The most common comorbidity reported was hypertension (n = 36, 51.4%), followed by dyslipidemia (n = 16, 22.8%) and diabetes (n = 11, 15.7%). Five (4.5%) patients reported an HIV infection, three (2.7%) a syphilis diagnosis (2.7%), and two (1.8%) a hepatitis C virus infection. Five (4.5%) patients reported a previous cancer diagnosis (one renal cancer, two skin cancer, and two Kaposi’s sarcoma). Full self-reported diagnoses can be seen in Supplementary Table S2. Fifty-one (46.4%) patients reported the daily use of prescribed medication, with an average of three different medications. Most of them reported the use of more than one medication category (51%), with the most used medications being antihypertensives (56.9%), diuretics (25.5%), lipid-lowering agents (21.6%), and antidiabetic/hypoglycemic agents (21.6%) (Supplementary Table S3).

### 3.5. Anthropometric, Pulse Oximetry, Blood Pressure and Body Temperature Measurements

The mean and standard deviation (SD) of the body mass index were 23.86 (±5.56), mean (SD). The mean BP was 125.65 (±25.58) × 84.46 (±14.72) mmHg. Only 14.5% of patients presented BP measurements within the normal range. No patients had abnormal body temperature measurements or a fever. The mean (SD) HR was 81.01 (±17.48), and the mean O_2_ saturation was 95.90 (±2.31).

### 3.6. Laboratory Tests

The laboratory results are summarized in Table 2. The following tests present a higher percentage of altered results: CRP (63.6%), hemoglobin (60%), erythrocytes (57.3%), hematocrit (59.1%), gamma-glutamyl transferase [GGT] (45.5%), 25-Hydroxy vitamin D (47.3%), RDW-SD (36.4%), neutrophil counts (35.5%), eosinophil counts (35.5%), total cholesterol (35.5%), high-density lipoprotein (HDL) (35.5%), iron (34.5%), and glucose (34.5%). Overall, 11 (10%) of the included patients reported systemic infectious diseases at baseline. Although only three (2.7%) patients reported a syphilis diagnosis, nine (8.2%) additional individuals tested positive for syphilis; thus, a total of 12 (10.9%) diagnoses of syphilis were observed in this study.

### 3.7. Treatment-Related Head and Neck Toxicities

Sixty-three (57.3%) patients underwent full curative treatment (i.e., RT or CRT, either following previous surgery or without prior surgery).

Progressive toxicities, including OM, xerostomia, dysphagia, dysgeusia, trismus, and radiodermatitis, were observed irrespective of the selected radiation therapy modality, whether it was intensity-modulated radiation therapy (IMRT) or three-dimensional (3D). There was a positive correlation between the number of medications in use and OM (0.268) and a negative correlation between the number of medications in use and dysgeusia (−0.257) outcomes. There were no correlations between the number of diagnosed comorbidities and toxicities. Full correlation values are shown in Table 3.

The association between altered laboratory exam results performed prior to oncological treatment on days 5/10/15/20/25/30 and 33–35 of treatment-related toxicities (OM, dysgeusia, dysphagia, xerostomia, radiodermatitis, trismus, and OC) can be seen in Table 4. In the OM domain, altered thyroxine (T4) and free thyroxine (FT4) had significant *p* values (*p* < 0.05) starting on D15; calcium levels were altered on D5/10/30; urea levels were altered on D25/30/D33–35; creatinine levels were altered on D15/30; alkaline phosphatase levels were altered on D20/25.

A positive syphilis diagnosis was correlated with mucositis on D15/30. OC was associated with syphilis and HIV on D20. Table 4 presents the full association between altered laboratory exams and treatment-related H and N toxicities.

When evaluating Table 4 for laboratory test exams that were altered and correlated with toxicity on more than four different evaluation days with significant *p* values (*p* < 0.05), it is observed that altered T4 and FT4 appear to be correlated with mucositis. Accordingly, a correlation between T4 and mucositis is seen on D20 (*p* = 0.0234) and D25 (*p* = 0.0281), with a slightly lower correlation on D30 (*p* = 0.0718) and D33–35 (*p* = 0.0503). The correlation between OM and FT4 is only significant on D33–35 (*p* = 0.0426) but is close on D20 (*p* = 0.0519), D25 (*p* = 0.0958), and D30 (*p* = 0.0875) (Table 5).

**Figure 1:**
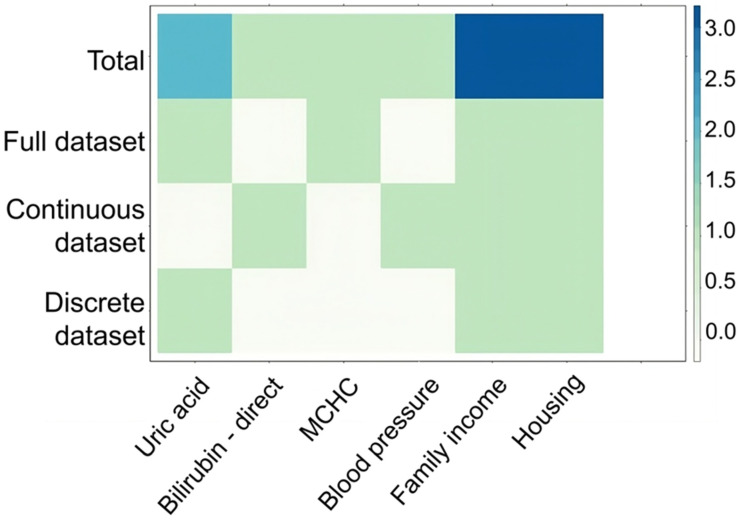
Heat map of oral mucositis predictors. The image above depicts the oral mucositis predictors based on our total, full, continuous, and discrete datasets. The predictors were tested across all the data in this investigation. The most important factors are family income and housing.

The most important predictors for OM were family income and housing; both outcomes are presented in navy blue on the heat map (Figure 1).

### 3.8. OHIP-14

The mean OHIP-14 score from the study population was 19.5, ranging from 0 to 49. The worst domain reported was physical pain (4.37 out of possible 8), which evaluated pain and difficulty in eating (Supplementary Table S4).

## 4. Discussion

This study presents original insights into the systemic alterations in patients with OOPSCC who undergo dental treatment before starting cancer therapy and their medical predictors of treatment-related oral toxicities. The results indicate that, despite the heightened prevalence of comorbidities and abnormal laboratory results in this patient population, dental treatment prior to cancer treatment did not result in any adverse events, including bleeding and infection. Significantly, there exists clinical importance in employing distinct laboratory parameters such as T4, FT4, urea, creatinine, calcium, alkaline phosphatase, and a positive syphilis diagnosis as predictive indicators for the onset of OM and OC during the course of cancer treatment. These findings emphasize the need for a nuanced approach in managing oral health during cancer treatment and suggest new opportunities for developing tailored systemic care protocols to mitigate the risk of oral toxicities in OOPSCC patients.

This study operationalized the term “comorbidity” to denote coexisting disease processes unrelated to the primary disease under investigation [19]. Consistent with the findings presented herein, the current literature underscores the heightened prevalence of comorbidities among patients with OOPSCC compared to the general population, primarily attributed to chronic smoking and alcohol exposure [20,21]. A comprehensive review reported that approximately 60% of H and N cancer patients experience concurrent illnesses [19]. Notably, our investigation, initially focused on an advanced OOPSCC cohort in Latin America, revealed that 85.4% of patients had a history of tobacco/alcohol consumption. Moreover, patients reported an overall comorbidity rate of 63.6%, with 44.3% having one concurrent illness, and 55.7% experiencing two or more concurrent conditions.

The most common comorbidities reported were hypertension (51.4%), followed by dyslipidemia (22.8%) and diabetes (15.7%). This is similar to the results of a study population of 10,524 H and N cancer patients (3049 diagnosed with oral cancer, and 2499 diagnosed with oropharyngeal cancer), with the most reported comorbidities being hypertension (59.6%), hyperlipidemia (31.4%), chronic obstructive pulmonary disease (COPD; 26.4%), and diabetes (21.1%) [22]. Comorbidities can impact the diagnosis, prognosis, survival, and treatment of patients with cancer, dictate the cancer treatment modality, and shape the way dental treatment is provided [22,23].

The most frequent altered laboratory findings were elevated CRP, altered levels of hemoglobin, erythrocytes, hematocrit, GGT, 25-Hydroxy vitamin D, RDW-SD, neutrophil counts, eosinophil counts, total cholesterol, HDL, iron, and glucose. A retrospective study involving 261 H and N cancer patients, which evaluated pre-therapeutic laboratory values, also demonstrated that elevated CRPs were the most frequent laboratory anomaly (60%), but they also observed impaired liver enzymes (30–50%), leukocytosis (20%), and anemia (10%) [24]. 

CRP is a nonspecific inflammation marker synthesized in response to acute inflammation or destruction of tissue cells, and over-expressed levels are demonstrated to be prognostic markers in various tumors, including lung, lymphoma, and, more recently, H and N cancers [25]. Altered liver function GGT can be explained by chronic alcohol abuse in this population [25]. The assessment of 25-Hydroxy vitamin D serves as a key measure for monitoring vitamin D levels, with deficiency being markedly prevalent among adults. Existing evidence has associated low vitamin D levels with conditions such as hypertension, cancer, and diabetes mellitus [26,27]. Despite these associations, establishing a definitive causal link between HNC and vitamin D levels remains inconclusive. A comprehensive literature review suggests a potential inverse relationship between the risk of HNC and vitamin D levels; however, given the complexity of this association and the existing gaps in current knowledge, further studies are necessary to elucidate any correlation between decreased vitamin D levels and an elevated risk of HNC [26,27]. Neutrophils, the most abundant leukocytes in the blood, are the first line of defense during inflammation and infections. High counts in the H and N cancer population are associated with poor cancer prognosis. Low counts are associated with infection [26,27,28]. The high incidence of altered blood glucose concentration is associated with the high number of diabetic patients in this targeted sample.

The existing literature indicates that general dentists might possess limited experience in managing cancer patients. Typically, professionals specializing in oral oncology are responsible for diagnosing and managing oral conditions and diseases in patients with OOPSCC [29]. While this holds true for individuals currently undergoing or having completed cancer treatment, our study implies that routine dental procedures before the onset of OOPSCC may be effectively carried out by general dentists. Our findings indicate that, notwithstanding the elevated prevalence of comorbidities and abnormal laboratory results, receiving dental treatment prior to cancer therapy necessitates no modifications and poses no complications associated with dental procedures.

To the best of our knowledge, this study represents the inaugural attempt to establish correlations among the presence of comorbidities, laboratory alterations, the administration of routine dental care preceding the initiation of cancer therapy, and the subsequent occurrence of toxicities during curative treatments (RT or CRT) in patients with OOPSCC. Due to the originality of our findings, there is scant existing literature available to either substantiate or compare the majority of our results. Treatment toxicities in H and N cancer are not only prevalent but also substantially impact the patients’ quality of life, potentially leading to treatment interruptions, with adverse effects on prognosis [3,30]. Within the scope of our study, we found that toxicities such as OM, xerostomia, dysphagia, dysgeusia, trismus, and radiodermatitis exhibited a progressive trajectory over time, irrespective of the chosen RT modality. Remarkably, we identified a positive correlation between the number of medications in use and the incidence of OM, while a negative correlation was observed between the number of medications and outcomes related to dysgeusia.

Some findings in the present study may also be considered predictive of oral toxicities during oncological treatment. We demonstrated that urea levels were altered on D25/30/D33–35, and creatinine levels were affected on D15/30 of RT or CRT treatment, pointing to altered renal function and impairment of drug metabolism in patients undergoing these treatment modalities. Ultimately, this could imply a more severe form of mucositis [29]. Additionally, we observed that altered T4 and FT4 levels were correlated with OM. Altered T4 levels and OM were seen on D20/25, with a slightly less impact on D30/33–35. The correlation between OM and FT4 was only significant on D33–35, but also showed a trend on D20/25/30. 

A syphilis diagnosis was correlated with OM on D15, and OC was associated with syphilis and HIV diagnoses on D20 of the RT treatment. The baseline immunosuppression, added to the cancer treatment-related immunosuppression, might favor this scenario [31]. The observation that syphilis infection may serve as a potential clinical predictor for the development of oral mucositis in patients undergoing chemoradiotherapy for oral and oropharyngeal cancer is an intriguing finding in the present study. While it may be premature to advocate for its immediate inclusion in a protocol for assessing medical conditions before the initiation of cancer treatment, this original result does have merit and warrants further investigation. This study, which included a comprehensive investigation of infectious diseases in its protocol, identified syphilis as the sole infection showing potential as a risk marker for oral mucositis. It is important to acknowledge the limitations inherent in establishing a cause-and-effect relationship within the limits of this study. However, the clinical observation is noteworthy, especially considering the chronic and multi-stage nature of syphilis. In the broader context of evaluating medically complex patients before the onset of cancer treatment, the inclusion of syphilis is particularly significant, considering its resurgence in various nations, posing a global public health concern [32]. While cautious interpretation is warranted, the originality of this result highlights the need for validation through larger prospective clinical studies, paving the way for a more comprehensive understanding of the potential role of syphilis as a predictor of adverse effects development during cancer treatment.

Notably, both family income and housing emerged as significant predicting factors for OM. The observed influence of these predictors can be contextualized within the sociodemographic landscape of OOPSCC patients in Brazil. The correlation between low income and education levels and their potential impact on patient compliance and adherence to recommendations may exacerbate the severity of OM grades [30]. In our study population, the mean OHIP-14 score was 19.5, with the most adversely affected domain being physical pain (4.37). These results parallel a study conducted in Brazil involving H and N cancer patients, where the mean OHIP-14 score was 19.52 (±11.79), with physical pain (3.70 ± 2.44) identified as the primary factor affecting QoL outcomes [30]. Together, these findings underscore the consistent significance of socioeconomic factors and oral health impacts, emphasizing their relevance in shaping the QoL outcomes among OOPSCC patients in Brazil.

The present study has limitations that warrant consideration. Firstly, the relatively short follow-up time post-diagnosis and treatment prevents a direct correlation between abnormal laboratory test values and the prognosis or treatment outcomes of patients with OOPSCC. Furthermore, the study employs diverse treatment modalities, adding complexity to the interpretation of results. Additionally, certain observed correlations impose further limitations on the generalizability of the findings.

In conclusion, this study suggests that despite the elevated prevalence of comorbidities and abnormal laboratory results in this patient population, dental treatment performed prior to OOPSCC therapy exhibits no adverse events, such as bleeding or infection. Noteworthy is the clinical relevance of specific laboratory findings, including T4, FT4, urea, creatinine, calcium, alkaline phosphatase, and a positive syphilis diagnosis, as predictive indicators for the development of OM and OC during cancer treatment. When taken together, these results highlight the potential clinical relevance of incorporating laboratory findings as predictive indicators for cancer treatment-related oral toxicities among patients with OOPSCC. Considering these outcomes, a compelling need to explore the integration of a systemic care protocol before initiating cancer treatment emerges to mitigate the risk of oral toxicities. Such an approach could involve targeted interventions addressing identified risk factors, potentially improving patient outcomes. Future studies could explore deeper into refining and validating such systemic care protocols, assessing their feasibility and cost-effectiveness, as well as elucidating their long-term impact on oral health outcomes in the context of OOPSCC treatment.

## Figures and Tables

**Table 1:** Sociodemographic, clinicopathological, oncologic treatment, and referral to dental treatment characteristics of the included patients (n = 110).

Variable	Value
Number of patients	110
Age, mean ± SD [range], years	57.32 ± 9.74 [23–87]
Sex, no. (%)	
Female	26 (23.6)
Male	84 (76.4)
Ethnicity, no. (%)	
White	48 (43.6)
Black	13 (11.8)
Yellow Brown	1 (0.9) 48 (43.6)
Cancer diagnosis, no. (%)	
Oral cavity	50 (45.5)
Oropharynx	60 (54.5)
Stage, no. (%)	
In situ	1 (0.9)
I	10 (9.1)
II	11 (10)
III	22 (20)
IVa/b/c	63 (57.3)
Not specified	3 (2.7)
p16 in oropharynx cases, no. (%)	
Negative	18 (30)
Positive	16 (26.7)
Not specified	26 (43.3)
Smoker (current or past), no. (%)	94 (85.4)
Alcohol consumption (current or past), no. (%)	94 (85.4)
Drug use (current or past) no. (%)	3 (2.7)
Education	
No education or less than 1 year	6 (5.5)
1 to 3 years	4 (3.6)
4 to 7 years	42 (38.2)
8 to 10 years	13 (11.8)
11 to 14 years	25 (22.7)
15 or more years	20 (18.2)
Marriage	
Single	28 (25.5)
Married/partnered	46 (41.8)
Separated/divorced	22 (20)
Widowed	14 (12.7)
Housing	
Own	72 (65.4)
Rent	26 (23.6)
Short-term	12 (11)
Average monthly income *	
<1 minimum wage	32 (29.1)
1 minimum wage	38 (34.5)
2 to 4 minimum wages	33(30)
5 or + minimum wages Average family monthly income *	7 (6.4)
<1 minimum wage	13 (11.8)
1 minimum wage	30 (27.3)
2 to 4 minimum wages	55 (50)
5 or + minimum wages	12 (10.9)
Cancer treatment	
Curative	82 (75.5)
S	18 (22)
S + induction CT + CRT	1 (1.2)
S + CRT	18 (22)
Induction CT + CRT	3 (3.6)
RT	17 (20.7)
CRT	25 (30.5)
Palliative	28 (25.5)
Referral	
Before radiotherapy	80 (72.8)
During chemotherapy	3 (2.7)
Before surgery	27 (24.5)

Abbreviations: no., total number of patients; %, percentage; SD, standard deviation; S, surgery; CT, chemotherapy; CRT, chemoradiotherapy; RT, radiotherapy. * National minimum wage in Brazil equals 1212 BRL/month (approximately 235 USD—2022 values).

**Table 2:** The laboratory results, percentage altered, range, mean, and standard deviation.

Laboratory Tests	n (%) Altered	Mean	SD
Complete blood count (CBC)			
Erythrocytes (×10^6^/mm^3^)	63 (57.3)	4.26	0.73
Hemoglobin (g/dL)	66 (60)	12.60	1.91
Hematocrit (%)	65 (59.1)	37.66	5.51
MCV (fL)	20 (18.2)	88.64	6.07
MCH (pg)	13 (11.8)	29.66	2.34
MCHC (g/dL)	9 (8.2)	33.45	1.06
RDW-CV (%)	15 (13.6)	13.33	1.25
RDW-SD (fL)	40 (36.4)	43.00	4.65
Erythroblasts (%)	1 (0.9)	0.1	0.07
Platelets (×10^3^/mm^3^)	25 (22.7)	325.57	115.38
MPV (fL)	30 (27.3)	10.20	1.10
Leukocytes (10^3^/mm^3^)	27 (24.5)	9.09	4.10
Neutrophils (10^3^/mm^3^)	39 (35.5)	6.66	6.64
Eosinophil (10^3^/mm^3^)	39 (35.5)	0.26	0.28
Basophil (10^3^/mm^3^)	27 (24.5)	0.05	0.05
Lymphocytes (10^3^/mm^3^)	37 (33.6)	1.93	0.72
Monocytes (10^3^/mm^3^)	35 (31.8)	0.73	0.30
Basic electrolyte panel			
Sodium (mEq/L)	12 (10.9)	138.66	3.30
Potassium (mEq/L)	17 (15.5)	4.53	0.45
Magnesium (mg/dL)	4 (3.63)	2.01	0.22
Chloride (mEq/L)	4 (3.6)	2.01	0.22
Iron (μg/dL)	38 (34.5)	76.43	34.15
Creatinine (mg/dL)	27 (24.5)	0.87	0.24
Glucose (mg/dL)	38 (34.5)	96.94	21.41
Urea (mg/dL)	11 (10)	34.72	15.69
Metabolic panel			
Calcium (mg/dL)	21 (19.1)	9.94	1.05
Bilirubin total (mg/dL)	11 (10)	0.36	0.16
Bilirubin direct (mg/dL)	7 (6.4)	0.18	0.07
Bilirubin indirect (mg/dL)	25 (22.7)	0.18	0.11
Alkaline phosphatase (U/L)	12 (10.9)	85.68	31.73
Aspartate aminotransferase (U/L)	6 (5.5)	19.53	7.67
Gamma-glutamyl transferase (U/L)	50 (45.5)	83.99	91.09
Alanine aminotransferase (U/L)	14 (12.7)	21.28	14.07
Lipid panel			
Total cholesterol (mg/dL)	39 (35.5)	183.05	42.06
High-density lipoprotein (HDL) (mg/dL)	39 (35.5)	45.81	12.18
Low-density lipoprotein (LDL) (mg/dL)	12 (10.9)	114.15	35.89
Non-high-density lipoprotein (non-HDL) cholesterol (mg/dL)	26 (23.6)	138.39	40.82
VLDL (very-low-density lipoprotein) (mg/dL)	17 (15.5)	24.81	10.76
Triglycerides (mg/dL)	31 (28.2)	136.63	80.30
Thyroid function			
Thyroid stimulating hormone (TSH) (μL/mL)	15 (13.6)	2.56	2.19
Total triiodothyronine (T3) (ng/dL)	12 (10.9)		
Thyroxine (T4) (μg/dL)	2 (1.8)	8.66	1.77
Free thyroxine (free T4) (ng/dL)	6 (5.45)	1.27	0.22
Glycated hemoglobin (hemoglobin A1c) (%)	14 (22.7)	5.35	0.80
Coagulation assay			
Prothrombin time (PT) (s)	2 (1.8)	14.43	1.20
International normalized ratio (INR) (s)	28 (25.5)	1.07	0.13
Activated partial thromboplastin time (aPTT) (s)	12 (10.9)	29.83	3.19
25-Hydroxy vitamin D (ng/mL)	52 (47.3)	26.86	14.41
HBsAg (hepatitis B surface antigen)	0		
Anti-HBs (hepatitis B surface antibody)	18 (16.4)	-	-
Anti-HBc (hepatitis B core antibody)	9 (8.2)	-	-
Hepatitis C	3 (2.7)	-	-
HIV	5 (4.5)	-	-
Syphilis	12 (10.9)	-	-
C-reactive protein (CRP) (mg/L)	70 (63.3)	23.43	31.94

Abbreviations: SD, standard deviation; %, percentage.

**Table 3:** Correlation analysis between the number of medications in use and the number of diagnoses of treatment-related toxicities.

	Pearson Correlation Coefficient	
	Number of Medications	Number of Diagnoses
Treatment-related toxicity		
Oral mucositis	0.268 *	0.101
Xerostomia	−0.043	0.233
Dysphagia	−0.183	−0.033
Dysgeusia	−0.257 *	−0.056
Trismus	0.031	0.082
Radiodermatitis	0.113	−0.017
Candidiasis	0.119	0.076

* Statistically significant association.

**Table 4:** Treatment side effects and association with abnormal laboratory results.

	Laboratory Exam			
Treatment Side Effects	Oral Mucositis	Dysgeusia	Dysphagia	Xerostomia
D5	FT4 (*p* = 0)	Erythroblasts (*p* = 0.007)	Sodium (*p* = 0.019)	Sodium (*p* = 0.019)
	Calcium (*p* = 0.002)	Hepatitis C (*p* = 0.007) Aspartate aminotransferase (*p* = 0.013) Alanine aminotransferase (*p* = 0.001) 25-Hydroxy Vitamin D	Basophil Hematocrit (*p* = 0.012) Erythrocytes (*p* = 0.002) Anti-HBc Hep B (*p* = 0.008) Cholesterol (*p* = 0.05) Calcium (*p* = 0.026)	Potassium (*p* = 0.003) Monocyte (*p* = 0.042) VLDL (*p* = 0.033) Cholesterol (*p* = 0.023)
D10	Leukocytes Hematocrit (*p* = 0.0058) Iron (*p* = 0.011) Calcium (*p* = 0.023)	Syphilis (*p* = 0.012)	Sodium (*p* = 0.044) MCH (*p* = 0.043) Hematocrit (*p* = 0.006) Erythrocyte (*p* = 0.001) Anti-HBc Hep B (*p* = 0.019) Gamma-glutamyl transferase (*p* = 0.027)	FT4 Syphilis (*p* = 0.02)
D15	aPTT (*p* = 0.019) FT4 (*p* = 0.035) T4 (*p* = 0.001) T3 (*p* = 0.001) Syphilis (*p* = 0.038) Creatinine (*p* = 0)	Bilirubin (*p* = 0.01)	FT4 (*p* = 0) T4 (*p* = 0.041) Eosinophil (*p* = 0.003) Hematocrit (*p* = 0.004) Erythrocytes (*p* = 0.003) Glucose (*p* = 0.045) Calcium (*p* = 0.011) Alkaline phosphatase (*p* = 0.028)	FT4 (*p* = 0.04) Lymphocyte (*p* = 0) VLDL (*p* = 0.041) Cholesterol (*p* = 0) Uric acid (*p* = 0.034)
D20	FT4 (*p* = 0) T4 (*p* = 0.045) Basophil (*p* = 0) Eosinophil (*p* = 0.046) non-HDL Cholesterol (*p* = 0.031) Glucose (*p* = 0) Alkaline phosphatase (*p* = 0.002)	TSH (*p* = 0.030) Basophil (*p* = 0.029) Hematocrit (*p* = 0.006) Erythroblasts (*p* = 0.005)	Potassium (*p* = 0) MCHC (*p* = 0.001) Hematocrit (*p* = 0.023) Erythrocyte (*p* = 0.018) Aspartate aminotransferase (*p* = 0)	Hepatitis (*p* = 0.029) HIV (*p* = 0)
D25	Urea (*p* = 0.008) FT4 (*p* = 0) T4 (*p* = 0.004) Monocytes (*p* = 0.036) Basophil (*p* = 0.004) Glucose (*p* = 0.001) Alkaline phosphatase (*p* = 0.002)	Hematocrit (*p* = 0.017) Erythrocytes (*p* = 0.020)	Neutrophil (*p* = 0.049) Hematocrit (*p* = 0.001) Erythrocyte (*p* = 0.002)	
D30	Urea (*p* = 0.001) FT4 (*p* = 0) T4 (*p* = 0) Syphilis (*p* = 0.018) Glycated hemoglobin (*p* = 0.030) Creatinine (*p* = 0.007) Chloride (*p* = 0.039) Calcium (*p* = 0.044) Bilirubin (*p* = 0.035)	Basophil (*p* = 0.008) Eosinophil (*p* = 0.019) Leukocyte (*p* = 0.048) Hematocrit (*p* = 0.025) Erythrocytes (*p* = 0.046)	Prothrombin time (*p* = 0.028) T4 (*p* = 0.028) Magnesium (*p* = 0) Neutrophil (*p* = 0.018) MCHC (*p* = 0.034) Hematocrit (*p* = 0.007) Erythrocyte (*p* = 0.023) Anti-HBc Hep B (*p* = 0.023)	Sodium (*p* = 0.025)
D33/35	Urea (*p* = 0) FT4 (*p* = 0.02) T4 (*p* = 0.009) Hematocrit	RDW-SD (*p* = 0.027) Hematocrit (*p* = 0.016) Creatinine	T4 Magnesium (*p* = 0.001)	T4 Sodium (*p* = 0.008) Glycated hemoglobin
	**Laboratory Exam**			
**Treatment Side Effects**	**Radiodermatitis**		**Trismus**	**Candidiasis**
D5	Prothrombin time (*p* = 0.024) Erythroblasts (*p* = 0.001) 25-Hydroxy Vitamin D (*p* = 0.002)		aPTT (*p* = 0) RDW-SD (*p* = 0.003) MCV (*p* = 0.001) HIV (*p* = 0.044) Anti-Hep B (*p* = 0.027)	MCHC (*p* = 0.009) Triglycerides (*p* = 0.047) Aspartate aminotransferase (*p* = 0.002)
D10	MCH (*p* = 0.014) Erythrocyte (*p* = 0.021) Calcium (*p* = 0.049)		aPTT (*p* = 0) RDW-SD (*p* = 0.001) Anti-Hep B (*p* = 0.026)	Potassium (*p* = 0.015) Alanine aminotransferase (*p* = 0.035) Potassium (*p* = 0)
D15	MCH (*p* = 0.014)		aPTT (*p* = 0) RDW-SD (*p* = 0.004) MCV (*p* = 0.001) Anti-HBc Hep B (*p* = 0.026)	Potassium (*p* = 0.05) Eosinophil (*p* = 0.032) Triglycerides (*p* = 0.035) Calcium (*p* = 0.039)
D20	INR (*p* = 0.006) Sodium (*p* = 0) MCH (*p* = 0.013) MCV (*p* = 0) Chloride (*p* = 0.017) Uric acid (*p* = 0)		RDW-SD (*p* = 0.004) MCV (*p* = 0.001) Anti-HBc Hep B (*p* = 0.026) aPTT (*p* = 0)	aPTT (*p* = 0.025) TSH (*p* = 0.015) Syphilis (*p* = 0.038) Eosinophil (*p* = 0.016) HIV (*p* = 0.018) Calcium (*p* = 0.011)
D25	INR (*p* = 0.017) Lymphocyte (*p* = 0.004) Non-HDL (*p* = 0.02) Cholesterol (*p* = 0.009) Chloride (*p* = 0)		aPTT (*p* = 0) RDW-SD (*p* = 0.002) MCV (*p* = 0.001) Hematocrit (*p* = 0.012) Erythrocyte (*p* = 0.012) Anti-HBc hep B (*p* = 0.023) Alkaline phosphatase (*p* = 0.042)	FT4 (*p* = 0.02) Eosinophil (*p* = 0.03) Glucose (*p* = 0.04) Alanine aminotransferase (*p* = 0.019)
D30	INR (*p* = 0.034) Potassium (*p* = 0) Lymphocyte (*p* = 0.013) Chloride (*p* = 0) Bilirubin Aspartate aminotransferase (*p* = 0.019)		aPTT (*p* = 0) RDW-SD (*p* = 0.002) VCM (*p* = 0.001) Hematocrit (*p* = 0.031) Erythrocytes (*p* = 0.033) Anti-HBc Hep B (*p* = 0.022)	Potassium (*p* = 0.024) Eosinophil (*p* = 0.039) Iron Calcium (*p* = 0.037)
D33/35	FT4 (*p* = 0.005) Potassium (*p* = 0) Aspartate aminotransferase (*p* = 0.01) Alkaline phosphatase		aPTT (*p* = 0) RDW-SD (*p* = 0.031) MCV (*p* = 0.007) Hematocrit (*p* = 0.008) Erythrocyte (*p* = 0.011) HDL cholesterol (*p* = 0.044) Iron (*p* = 0.050) Bilirubin (*p* = 0.026) Alkaline phosphatase (*p* = 0.039)	INR (*p* = 0.003) CRP (*p* = 0.026) Monocytes LDL (*p* = 0.041) Cholesterol (*p* = 0.038) Glucose (*p* = 0.005)

Abbreviations: aPTT: Activated partial thromboplastin time; CRP—C-reactive protein; D: days; HDL: high-density lipoprotein; Hep B—hepatitis B; HIV—human immunodeficiency virus; INR—international normalized ratio; LDL—low-density lipoprotein; MCH—mean corpuscular hemoglobin; MCV—mean corpuscular volume; MCHC—mean corpuscular hemoglobin concentration; RDW-SD—erythrocyte distribution width (standard deviation); TSH—thyroid stimulating hormone; T3—total triiodothyronine; T4—thyroxine; VLDL—very-low-density lipoprotein.

**Table 5:** Mucositis association with abnormal laboratory results observed four times or more on different evaluation days.

	D5	D15	D20	D25	D30	D35
T4						
R		−0.1570	−0.2830	−0.2768	−0.2266	−0.2703
IC95%		−0.3941 to 0.09973	−0.5000 to −0.03260	−0.4966 to −0.02371	−0.4534 to 0.02776	−0.5099 to 0.008176
P		0.2154	0.0234 *	0.0281 *	0.0718	0.0503
FT4						
R	0.07591	−0.1388	−0.2441	−0.2117	−0.2154	−0.2796
IC95%	−0.1824 to 0.3244	−0.3783 to 0.1181	−0.4680 to 0.009257	−0.4426 to 0.04553	−0.4440 to 0.03957	−0.5173 to −0.001844
P	0.5543	0.2740	0.0519	0.0958	0.0875	0.0426 *

* Statistically significant values. Abbreviation: D: day, T4: thyroxine, FT4: free thyroxine.

## Data Availability

The raw data supporting the conclusions of this article will be made available by the authors on request.

## Supplementary Materials

The following supporting information can be downloaded at: https://www.mdpi.com/article/10.3390/dj12040089/s1, Table S1: Laboratory tests and the reference values. Table S2: List of the comorbidities reported by the included patients (n = 110). Table S3: Medication in use data and the most used medication categories. Table S4: OHIP-14 questionnaire, mean score and standard deviation per answer, and mean total, range and standard deviation.
