# Supplementary material for: Prospective Cohort Study Identifies Medical Predictors of Treatment-Related Oral Toxicities in Oral and Oropharyngeal Cancer Patients
Source: Dent J (Basel). 2024 Apr 1;12(4):89. doi: 10.3390/dj12040089 (PMC11049634; doi:10.3390/dj12040089)
Supplement: Supplementary file 1 [file dentistry-12-00089-s001.zip › dentistry-2771382-supplementary.pdf]

Supplementary Table S1. Laboratory tests and the reference values.

Laboratory tests	Reference values
**Complete blood count (CBC)**	
Erythrocytes (x10^6/mm^3)	M: 4.5 - 6.5 / F: 3.9 – 5.6
Hemoglobin (g/dL)	M: 13.5 - 17.5 / F: 11.5 – 15.5
Hematocrit (%)	M: 40 – 52 / F: 36 – 48
MCV (fL)	80 – 95
MCH (pg)	27 – 34
MCHC (g/dL)	30 – 35
RDW-CV (%)	M: 11.6 - 14.4 / F: 11.7 – 14.4
RDW-SD (fL)	M: 35.1- 43.9 / F: 36.4 – 46.3
Erythroblasts (%)	0
Platelets (x10^3/mm^3)	150 – 400
MPV (fL)	9.4 - 12.4
Leukocytes (10^3/mm^3)	4 – 11
Neutrophils (10^3/mm^3)	2.5 – 7.5
Eosinophil (10^3/mm^3)	0.04 – 0.44
Basophil (10^3/mm^3)	0.00 – 0.1
Lymphocytes (10^3/mm^3)	1.5 – 3.5
Monocytes (10^3/mm^3)	0.2 – 0.8
**Basic electrolyte panel**	
Sodium (mEq/L)	135 – 145
Potassium (mEq/L)	3.5 – 5
Magnesium (mg/dL)	1.58 – 2.55
Chloride (mEq/L)	98 – 107
Iron (ug/dL)	M: 65 – 175 / F: 50 – 170
Creatinine (mg/dL)	M: 0.7 –1.2 / F: 0.5 – 0.9
Glucose (mg/dL)	70 – 99
Urea (mg/dL)	10 - 50
**Metabolic panel**	
Calcium (mg/dL)	12 - 60 years : 8.40 - 10.20 / 61 - 90 years: 8.60 - 10.20
Bilirubin total (mg/dL)	0.2 - 1.0
Bilirubin direct (mg/dL)	< 0.3
Bilirubin indirect (mg/dL)	0.10 - 0.60
Alkaline phosphatase (U/L)	M: 40 - 129 / F: 35 - 104
Aspartate aminotransferase (U/L)	M: < 37 / F: < 31

Gamma-glutamyl transferase (U/L)	M: 8 - 61 / F: 5 - 36
Alanine aminotransferase (U/L)	M: < 41 / F: < 31
**Lipid panel**	
Total cholesterol (mg/dL)	< 190
High-density lipoprotein (HDL) (mg/dL)	> 40
Low-density lipoprotein (LDL) (mg/dL)	Optimal: < 100, Near/ above optimal: 100 - 129, Borderline High: 130 - 159, High 160 – 189, and Very high >/ 190
Non-high-density lipoprotein (non-HDL) cholesterol (mg/dL)	Optimal: < 130, Acceptable: 130 – 159; High: 160 – 189, Very high: >/ 190
VLDL (very-low-density lipoprotein) (mg/dL)	< 35
Triglycerides (mg/dL)	< 150
**Thyroid function**	
Thyroid stimulating hormone (TSH) (ul/mL)	0.27 – 4.20
Total triiodothyronine (T3) (ng/dL)	80 – 200
Thyroxine (T4) (ug/dL)	5.1 - 14.1
Free thyroxine (free T4) (ng/dL)	0.93 - 1.7
**Glycated hemoglobin (Hemoglobin A1C)** (%)	4.1 – 6
**Coagulation assay**	
Prothrombin time (PT) (s)	10.3 - 16.6
International Normalized Ratio (INR) (s)	0.95 - 1.20
Activated partial thromboplastin time (aPTT) (s)	25.4 - 36.9
25-Hydroxy Vitamin D (ng/mL)	< 60 years old: >20 / > 60 years old: 30 - 60
HBsAg (Hepatitis B surface antigen)	reactive or not reactive
Anti-HBs (Hepatitis B surface antibody	reactive or not reactive
Anti-HBc (Hepatitis B core antibody)	reactive or not reactive
Hepatitis C	reactive or not reactive
HIV	reactive or not reactive
Syphilis	reactive or not reactive
C-reactive protein (CRP) (mg/L)	<5.0 mg/L

Abbreviations: MCV - mean corpuscular volume; MCH - mean corpuscular hemoglobin; MCHC - mean corpuscular hemoglobin concentration; RDW - Erythrocyte Distribution Width; RDW-SD - Erythrocyte Distribution Width (standard deviation); MPV - mean platelet volume (MPV).

Supplementary Table S2. List of the comorbidities reported by the included patients (n = 110).

Diagnosis	Value n (%)
Hypertension	36 (51.4)
Dyslipidemia	16 (22.9)
Diabetes	11 (15.7)
Gastritis	9 (12.9)
HIV	5 (7.1)
Previous cancer	5 (7.1)
Acute myocardial infarction	5 (7.1)
Tuberculosis (treated)	5 (7.1)
Obesity	5 (7.1)
Asthma	3 (4.3)
Gout	3 (4.3)
Hypothyroidism	3 (4.3)
Impaired vision	3 (4.3)
Syphilis	3 (4.3)
Anxiety	2 (2.9)
Arrhythmia	2 (2.9)
Stroke	2 (2.9)
Bronchitis	2 (2.9)
Hepatic steatosis	2 (2.9)
Congestive heart failure	2 (2.9)
Prediabetes	2 (2.9)
Osteoporosis	2 (2.9)
Hepatitis C	2 (2.9)
Leg paresthesia	2 (2.9)
Arthritis	1 (1.4)
Chron disease	1 (1.4)
Coronary artery disease	1 (1.4)
Depression	1 (1.4)
Dyspepsia	1 (1.4)
Pulmonary emphysema	1 (1.4)
Migraine	1 (1.4)
Esophagitis	1 (1.4)
Glaucoma	1 (1.4)
Hemiparesis left	1 (1.4)
Hepatitis B	1 (1.4)
Spinal disc herniation	1 (1.4)
Parkinson investigation	1 (1.4)

Labyrinthitis	1 (1.4)
Osteoarthritis	1 (1.4)
Osteopenia	1 (1.4)
Hearing loss	1 (1.4)
Mitral valve prolapse	1 (1.4)
Gastroesophageal reflux	1 (1.4)
Poliomyelitis sequelae (leg)	1 (1.4)

Abbreviations: no., total number of patients; %, percentage.

Supplementary Table S3. Medication in use data and the most used medication categories.

Medication in use	Value n (%)
Yes	51 (46.4)
More than one medication category	26 (51)
Most used medication categories	
Antihypertensive	29 (56.9)
Diuretics	13 (25.5)
Lipid-lowering agents	11 (21.6)
Antidiabetic/ Hypoglycemic agents	11(21.6)
Proton pump inhibitors	7 (13.7)
Antiretroviral	5 (9.8)
Antidepressants	3 (5.9)
Gamma aminobutyric acid analogs	3 (5.9)
Laxatives	3 (5.9)

Abbreviations: n: number of patients; %: percentage.

Supplementary Table S4. OHIP-14 questionnaire, mean score and standard deviation per answer, and mean total, range and standard deviation.

**OHIP-14**<br>**Domain**	**Item**	**Mean (STDEV)**
Domain 1: Functional	1. Had trouble pronouncing any words?	1.41 (1,71)
	2. Felt sense of taste has worsened?	0.85 (1.49)
Domain 2: Physical pain	3. Had painful aching?	2.16 (1.69)
	4. Found it uncomfortable to eat any foods?	2.21 (1.86)
Domain 3: Psychological discomfort	5. Been self-conscious	2.29 (1.63)

	6. Felt tense	1.31 (1.56)
Domain 4: Physical disability	7. Felt diet has been unsatisfactory	2.31 (1.85)
	8. Had to interrupt meals	1.03 (1.43)
Domain 5: Psychological disability	9. Found it difficult to relax	1.36 (1.66)
	10. Been a bit embarrassed	0.57 (1.11)
Domain 6: Social disability	11. Been a bit irritable	0.59 (1.03)
	12. Had difficulty doing usual jobs	1.27 (1.64)
Domain 7: Handicap	13. Felt life less satisfying	1.62 (1.64)
	14. Been totally unable to function	0.52 (1.16)
	TOTAL (sum)	19.5 (12.5)
	Range	0 - 49

Abbreviations: OHIP-14 - The Oral Health Impact Profile.
